# Chest Pain With a Bizarre Electrocardiogram: Swiveling Around the Axis

**DOI:** 10.7759/cureus.24191

**Published:** 2022-04-16

**Authors:** Narut Prasitlumkum, Ramdas G Pai, Leenhapong Navaravong

**Affiliations:** 1 Cardiovascular Medicine, University of California Riverside, Riverside, USA; 2 Division of Cardiovascular Medicine, Department of Internal Medicine, University of Utah, School of Medicine, Salt Lake City, USA

**Keywords:** ecg, electrocardiogram, limb lead reversal, counterclockwise lead reversal, chest pain

## Abstract

Technical errors in electrocardiography acquisition can deviate from the correct diagnosis, ensuing in unnecessary workups and hospital billings. A keen understanding of lead placement concepts and the Einthoven triangle helps avoid these unwanted paths. Here, we presented the case of a 57-year-old woman with a history of hypertension and chronic kidney disease who came to the hospital due to chest pain. Initially, ischemic changes in her electrocardiogram (ECG) were noted. However, the correct placement of ECG leads confirmed the "counterclockwise lead placement" of this patient. This case report highlighted the underrecognized types of ECG lead reversals.

## Introduction

Lead misplacement is among the most common under-recognized problems, with a prevalence ranging from 0.4% to 4% in different circumstances [1]. Surprisingly, it can be misread by experienced cardiologists, as previously described in one anecdotal report [2]. Without a clear understanding of lead placement, a medical fallacy happens, which leads to a chain of mismanagement [3].

## Case presentation

**Figure 1:**
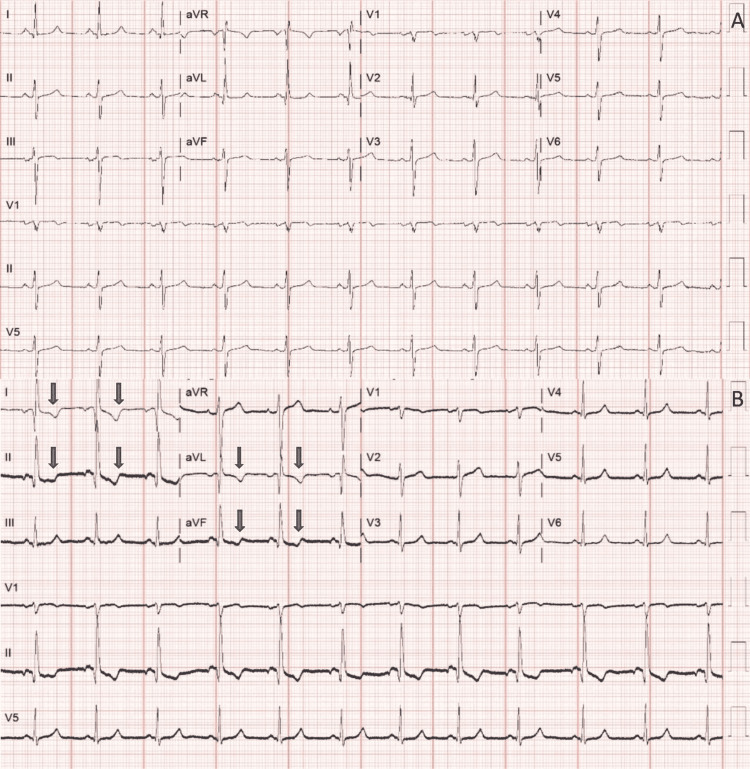
(A) Baseline 12-lead electrocardiogram at the initial encounter in emergency room; (B) a 12-lead electrocardiogram showing significant diffuse T-wave changes in limb lead (blue arrows).

A 57-year-old female presented to the emergency room (ER) due to chest pain accompanied by difficulty breathing. She had only a medical history of hypertension and chronic kidney disease. Initial evaluation by the ER demonstrated elevated fourth-generation troponin up to 0.077 µg/mL (upper limit ≤0.028 µg/mL). Computed tomography angiogram (CT-A) did not reveal a pulmonary embolus. Her initial electrocardiogram (ECG) revealed sinus rhythm with left anterior fascicular block, similar to her baseline weeks ago, as shown in Figure 1A. A repeat ECG was performed, showing a new T-wave inversion noted on leads I, II, aVL, and aVF (Figure 1B). The cardiology team was consulted for the possible acute coronary syndrome.

The second ECG (Figure 1B) showed a biphasic P wave in lead II and a negatively inverted P wave in leads I and aVL, in addition to the T-wave changes as described in the vignette. Strangely, there was an unusual positive P wave in lead aVR. The morphologies of QRS complexes in the second ECG (Figure 1B) aVR, aVL, and aVF were identical to baseline aVL, aVR, and aVF, respectively (Figure 1A). Precordial leads did not show any significant changes compared to the baseline ECG. Given the findings, this was consistent with a counterclockwise lead misplacement.

As the ECG shown in Figure 1B was considered a possible lead misplacement, the third repeat ECG was performed. This repeat ECG was similar to her ECG at her baseline (Figure 1A). On further questioning, her chest pain characteristics were also typical of musculoskeletal origin, presumably secondary to a recent fall hitting her chest onto the ground. A nuclear myocardial perfusion scan was ordered given her intermediate probability of acute coronary syndrome, which turned out to be an unremarkable perfusion study. Her troponin trended down from 0.077 to 0.06 µg/mL, corresponding to her improving creatinine level with hydration, from her initial value of 3.24 mg/dL decreasing to 2.95 mg/dL (her baseline). Later, her symptoms subsided with conservative management.

## Discussion

The most common, and possibly the easiest to recognize, lead malposition is right-left arm reversal [4], nimbly characterized by total inverted P and QRS waves in lead I and the transposition between leads aVR and aVL. Nonetheless, other uncommon patterns are more challenging, especially in the urgent and high-medical-demanded milieu, which leads to increased error rates [1].

Under normal circumstances, the sinus P wave should be upright in leads I, II, aVL, aVF, and left precordial leads (V2-V6) in consonance with normal atrial conduction polarity. On the contrary, either a negative or biphasic P wave can be present in lead III or aVF as their locations are opposite to the atrial electrical trajectory. For the same reason, the P wave in aVR should always be negative, as exhibited in Figure 1A. After the electrical conduction passes through the AV node, it conducts through the His bundle, Purkinje fibers, and ultimately ventricular tissues. This construes the QRS complex such that its absolute vector points toward the apex, normally at the axes from −30 to 90 [5]. In the view of precordial leads, a normal R-wave progression usually ascends from V1 to V6, establishing the rS complex on the right precordial leads while the Rs complex on the left precordial leads [6]. Knowing this principle can help decipher bizarre ECG features caused by this inadvertency. Conceivably, a distinct change from prior ECG, unexplained Q or R waves, the presence of mirroring or identical leads, and flat lines in the limb lead can serve as a clue to lead reversals [7].

**Figure 2:**
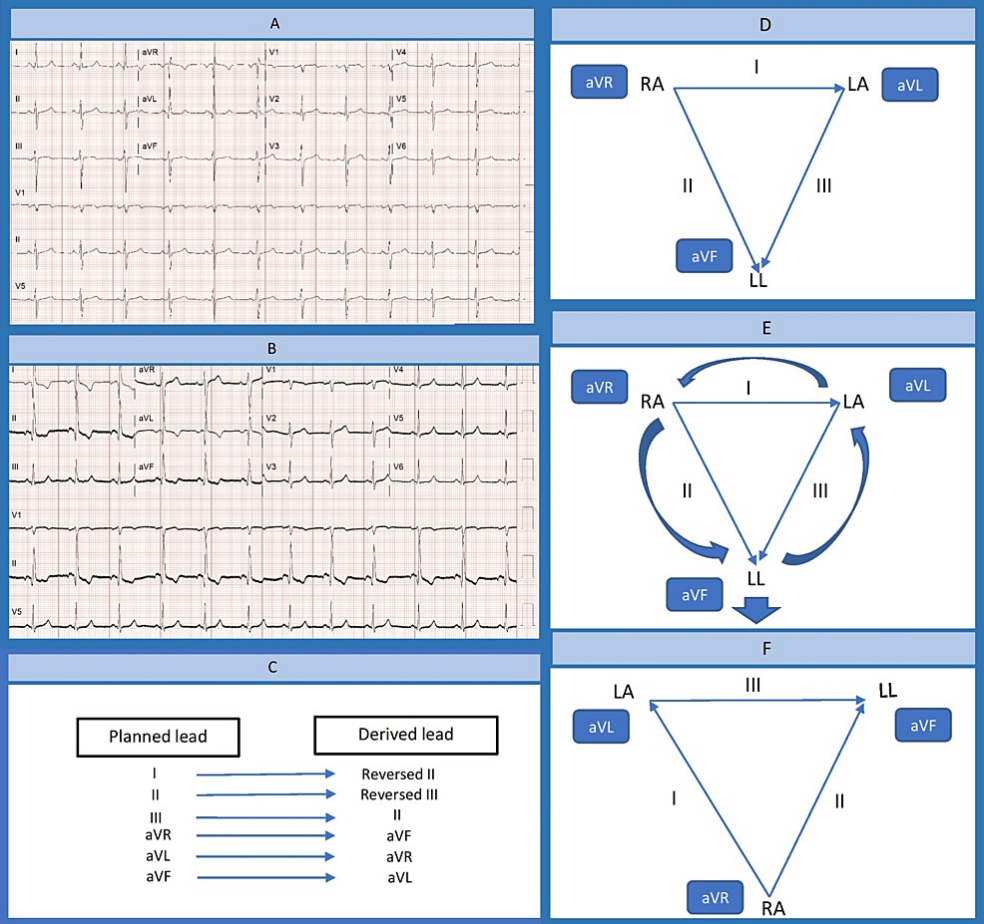
(A) A 12-lead electrocardiogram with normal lead placement; (B) a 12-lead electrocardiogram with counterclockwise rotation; (C) effect on ECG form counterclockwise rotation lead misplacement; (D) normal vector of the limb leads; (E) counterclockwise rotation of the limb leads; (F) changes in the vector after counterclockwise rotation aVF: augmented vector foot, aVL: augmented vector left, aVR: augmented vector right, LA: left arm, RA: right arm, LL, left leg

In our case, several findings argue against the normal configuration of lead placement. Considering the P wave depicted in Figure 1B, it violated its normalcy, hinting at the possibility of lead reversal. In augmented limb leads, the morphology of aVR was equivalent to the baseline aVF, aVF was equivalent to the baseline aVL, and aVL was equivalent to the baseline aVR. This feature raises the possibility of counterclockwise lead placement. As a result, abrupt changes in bipolar lead morphologies occur. The misplaced lead III has the same morphology as the baseline lead I as it falsely records a vector polarity from right to left arm. The misplaced lead II has an inverted appearance to the baseline lead III as it records a vector polarity from the left leg to the left arm. Lastly, the misplaced lead I has an inverted appearance of the baseline as the vector polarity from the left leg to the right arm is recorded (Figure 2).

In addition, significant ST-T wave changes in Figure 1B, compared to Figure 1A, were also secondary to counterclockwise lead placement. Broadly speaking, the presence of T-wave inversion is not pathognomonic of ischemia, though it is important to rule it out as several conditions can instigate this change, for example, electrolyte imbalance, normal variant, and extrasystoles and bundle branch blocks. Generally, T wave should be upright in leads I, II, and V3-V6 and always inverted in aVR [8]. Considering our showcase, T-wave changes were generalized in all limbs, which leads to a suspicion of diffuse ischemia that should be highly accountable until proven otherwise. Despite this possibility, it is deemed out of proportion as precordial ECG morphologies remain unchanged in Figure 1B in comparison to Figure 1A. Logically, there should be accompanying precordial ST-T changes, especially ST-segment elevation in aVR and V1, to suffice "circumstantial subendocardial ischemia" [9]. For this reason, T-wave changes, in this case, are non-physiologic and more consistent with lead malposition.

## Conclusions

Lead misplacement is not uncommon and can be easily mistaken. The counterclockwise lead reversal can be challenging to discern. The clue is the presence of an abnormal P-wave configuration with unchanged precordial ECG morphology. When there is a high suspicion, it is advisable to repeat the ECG and compare it with the baselines.
